# EcoTILLING for the identification of allelic variants of melon *eIF4E*, a factor that controls virus susceptibility

**DOI:** 10.1186/1471-2229-7-34

**Published:** 2007-06-21

**Authors:** Cristina Nieto, Florence Piron, Marion Dalmais, Cristina F Marco, Enrique Moriones, Ma Luisa Gómez-Guillamón, Verónica Truniger, Pedro Gómez, Jordi Garcia-Mas, Miguel A Aranda, Abdelhafid Bendahmane

**Affiliations:** 1 Centro de Edafología y Biología Aplicada del Segura (CEBAS)- CSIC, Apdo. correos 164, 30100 Espinardo, Murcia, Spain; 2 Unité de Recherche en Génomique Végétale (INRA-URGV), 2, rue Gaston Crémieux CP 5708, 91057 Evry Cedex, France; 3 Estación Experimental La Mayora (EELM)- CSIC, 29750 Algarrobo-Costa, Málaga, Spain; 4 Departament de Genètica Vegetal, Laboratori de Genètica Molecular Vegetal CSIC-IRTA, carretera de Cabrils s/n, 08348 Cabrils, Barcelona, Spain

## Abstract

**Background:**

Translation initiation factors of the 4E and 4G protein families mediate resistance to several RNA plant viruses in the natural diversity of crops. Particularly, a single point mutation in melon eukaryotic translation initiation factor 4E (eIF4E) controls resistance to *Melon necrotic spot virus *(MNSV) in melon. Identification of allelic variants within natural populations by EcoTILLING has become a rapid genotype discovery method.

**Results:**

A collection of *Cucumis *spp. was characterised for susceptibility to MNSV and *Cucumber vein yellowing virus *(CVYV) and used for the implementation of EcoTILLING to identify new allelic variants of *eIF4E*. A high conservation of *eIF4E *exonic regions was found, with six polymorphic sites identified out of EcoTILLING 113 accessions. Sequencing of regions surrounding polymorphisms revealed that all of them corresponded to silent nucleotide changes and just one to a non-silent change correlating with MNSV resistance. Except for the MNSV case, no correlation was found between variation of eIF4E and virus resistance, suggesting the implication of different and/or additional genes in previously identified resistance phenotypes. We have also characterized a new allele of *eIF4E *from *Cucumis zeyheri*, a wild relative of melon. Functional analyses suggested that this new *eIF4E *allele might be responsible for resistance to MNSV.

**Conclusion:**

This study shows the applicability of EcoTILLING in *Cucumis *spp., but given the conservation of eIF4E, new candidate genes should probably be considered to identify new sources of resistance to plant viruses. Part of the methodology described here could alternatively be used in TILLING experiments that serve to generate new *eIF4E *alleles.

## Background

Plant viruses are obligate parasites that infect plants owing to specific interactions between virus and host factors that determine the plant susceptibility to viral infection [1,2]. Mutation or loss of one such susceptibility factor may result in virus resistance. Therefore, genes encoding susceptibility factors constitute potential targets for biotechnological and genomics-assisted breeding for improvement of crops resistance to viruses [3]. Throughout the last decade several susceptibility factors to plant viruses have been identified and characterized using model organisms as experimental systems [4-6]. However, among these factors, only translation initiation factors of the 4E family (eIF4E and eIF [iso]4E) and eIF4 [iso]4G have been found to mediate resistance in the natural diversity of crops [6,7].

In the host cell, eIF4E is a part of the eIF4F protein complex, which has an essential role in the initiation step of cap-dependent mRNA translation. In eukaryotes, most cellular mRNAs contain terminal structures consisting of a 5'-cap and a 3'-poly(A) tail which are brought together through interactions with translation initiation factors to promote translation [8,9]. Significantly, positive-sense single stranded RNA viruses often lack the 5'-cap, the poly(A) tail or both of these structures, yet they need to use the host translational machinery to translate their mRNAs [10,11]. Indeed, mutagenesis of model hosts [12,13] and the characterization of some natural recessive resistance genes [14-22] have implicated eIF4E as a susceptibility factor required for plant virus multiplication.

Melon (*Cucumis melo *L.) is an economically important cucurbit crop cultivated in temperate, subtropical and tropical climates. It is a diploid species (2*n *= 2*x *= 24) which has an estimated genome size of 450 Mb. Virus resistance is a major melon breeding objective, as several diseases caused by viruses have great economical impact in melon crops worldwide. Significant examples include the cucumovirus (family *Bromoviridae*) *Cucumber mosaic virus *(CMV), the potyviruses (family *Potyviridae*)*Watermelon mosaic virus *(WMV), *Zucchini yellow mosaic virus *(ZYMV), the ipomovirus (family *Potyviridae*) *Cucumber vein yellowing virus *(CVYV), the crinivirus (family *Closteroviridae*)*Cucurbit yellow stunting disorder virus *(CYSDV) and the carmovirus (family *Tombusviridae*) *Melon necrotic spot virus *(MNSV) [23-25]. Despite this, not many natural resistance genes have been identified and introgressed into commercial melon cultivars. Probably, one of the most widely used is the *nsv *gene, which confers recessive resistance to all known strains of MNSV except to MNSV-264 [26]. There are at least two known sources of resistance to MNSV in melon: the cultivar Gulfstream and the Korean accession PI 161375, both controlled by *nsv *[27]. Recently, we have characterised the *nsv *locus demonstrating that it encodes melon eIF4E (Cm-eIF4E) and that a single amino acid change at position 228 of the protein leads to resistance to MNSV [18,28]. In this paper, we present the work done for the identification and characterization of new *nsv *alleles that could be responsible of resistance to MNSV. Thus, we screened a collection of *Cucumis *spp. accessions for MNSV susceptibility and analysed by EcoTILLING the diversity of *eIF4E *in this collection. EcoTILLING is a variation of TILLING (Targeting Induced Local Lesions in Genomes; [29]) which has been successfully used to examine genetic variation in *Arabidopsis *ecotypes [30] and wild populations of *Populus trichocarpa *[31]. We found a notable conservation of the exonic regions of *eIF4E *and showed that the only non-silent nucleotide change identified in *C. melo *accessions perfectly correlated with a phenotypic change in susceptibility to MNSV. Interestingly, a few accessions characterised in this work were previously identified as potential sources of resistance to viruses different than MNSV [32]. A comparison of data on virus susceptibility and variability in eIF4E suggested that other factors different than eIF4E are probably involved in these resistances. In addition, we have characterised a new *eIF4E *allele from *C. zeyheri *(*Cz-eIF4E*) which, in a functional analysis, appeared to be potentially responsible for the resistance of plants of this species to MNSV.

## Results

### Phenotyping *Cucumis *spp. accessions for virus susceptibility

We tested 135 *C. melo *and 12 wild relative accessions of the germplasm collection of Estación Experimental "La Mayora"- CSIC (Málaga, Spain) for their susceptibility to MNSV strains Mα5 (MNSV-Mα5, avirulent on melons of *nsv/nsv *genotype) [33] and 264 (MNSV-264, virulent on melons of the *nsv/nsv *genotype) [26] and to *Cucumber vein yellowing virus *(CVYV) [34]. Accessions were from different geographical origins: 3 from Africa, 7 from America, 17 from Central Asia, 90 from Europe (4 from Central Europe, 74 from Spain and 13 from other southern European regions), 3 from the Far East and India, 12 from Middle East and the remaining 14 from unknown origins (see Additional file 1).

**Figure 1:**
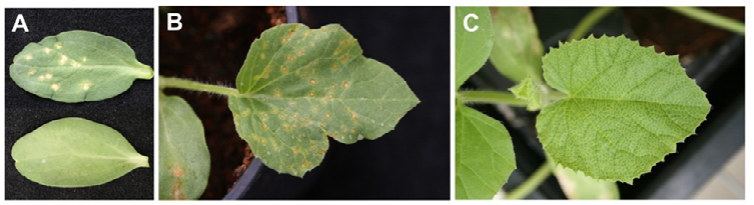
**Virus-induced symptoms in melon plants**. (*A*) Melon cotyledons inoculated with MNSV (top) and non-inoculated (bottom) at 7 days after inoculation. (*B*) A melon leaf showing systemic MNSV-induced symptoms at 14 days after inoculation. (*C*) A melon leaf showing systemic CVYV-induced symptoms at 12 days after inoculation.

Inoculations with MNSV showed that only one accession, C-277 (*C. zeyheri*), was resistant to both MNSV-Mα5 and MNSV-264. *C. melo *accessions C-178 and C-512, *C. dipsaceus *C-590, *C. meeusii *C-635 and *C. anguria *C-636 were resistant to MNSV-Mα5, but susceptible to MNSV-264. Symptoms on MNSV-inoculated cotyledons of susceptible accessions consisted of small necrotic lesions which appeared 4 to 5 days after inoculations (Figure 1A). Accessions *C. africanus *C-205 and C-633, *C. prophetarum *C-633, *C. ficifolius *C-637, *Cucumis spp*. C-753 and C-755, despite of being susceptible, showed a very low average number of virus-induced lesions per inoculated cotyledon (see Additional file 1). As reported by Mallor et al. [35], systemic symptoms appeared only in a proportion of the inoculated plants of susceptible accessions and consisted of small chlorotic spots in leaves that become necrotic a few days after appearance (Figure 1B), and necrotic streaks along the stems and petioles. The frequency of symptomatic plants varied with accessions. Moreover, a clear difference in the proportion of plants showing systemic symptoms after inoculation with MNSV-Mα5 (63%) and MNSV-264 (24%) was observed (see Additional file 1), suggesting that MNSV-Mα5 was more efficient than MNSV-264 in inducing systemic symptoms on mechanically inoculated plants.

Inoculations with CVYV showed that all *C. melo *accessions tested were susceptible, and that *C. africanus *C-205, *C. dipsaceus *C-588 and C-590 and *C. prophetarum *C-633 were resistant to this virus (see Additional file 1). No symptoms could be observed on inoculated cotyledons of all accessions, except for C-633. Systemic symptoms in susceptible accessions consisted of foliar mosaics and vein yellowing in young, newly emerged leaves which appeared about 10 to 12 days after inoculations (Figure 1C). Notably, resistance of C-633 plants was associated with the appearance of local necrotic lesions after CVYV mechanical inoculation (data not shown), suggesting an HR-like type of response.

### Screening of *eIF4E *polymorphisms by EcoTILLING

**Figure 2:**
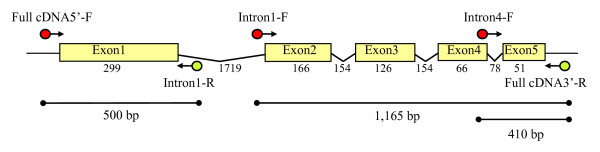
**Organization of *Cm-eIF4E *gene**. Exons are represented as boxes and the 5'UTR, 3'UTR and introns are shown as black broken lines (not to scale). Primers used in EcoTILLING are complementary to non-coding regions of the gene and are indicated by arrows. Amplified regions are represented by black lines. Sizes (bp) of PCR products are indicated below the lines. Sizes (bp) of exons and introns are also indicated.

**Figure 3:**
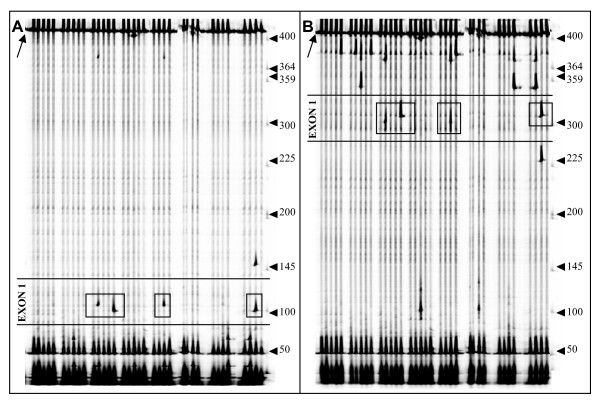
**Detection of polymorphisms in *Cm-eIF4E***. Gel images from the IRD700 (*A*) and IRD800 (*B*) channels of LI-COR analyzer. Each lane displays the 400 bp amplified product on Intron4-F/Full-cDNA3'-R primer combination digested with endonulcease *ENDO-I*. Heteroduplexes were produced after melting and annealing PCR products with the DNA of the reference genotype (cultivar Védrantais). A black arrow on the top left of each image indicates the position of homoduplex DNA. Arrows on the right of each panel indicate the molecular weight marker in bp. Cleaved products, indicated by boxes, correspond to sequence polymorphisms in exon 1. True polymorphisms should give rise to two complementary bands, one on each fluorescence channel.

In order to scan the complete coding region of *eIF4E *for natural sequence variation, three primer pairs to be used in EcoTILLING were designed on introns and on the 5' and 3' non-coding regions of the gene (Figure 2). Using these primers, we analysed a *Cucumis *spp. collection of 120 accessions previously characterised for their susceptibility to MNSV and CVYV (see above). Out of the 120 accessions, no PCR product was obtained from eight *C. melo *wild relative accessions, despite several attempts using different PCR amplification conditions. These eight accessions were thus excluded from further analysis. PCR products obtained from the remaining accessions were mixed with PCR products amplified from the cultivar Védrantais, which was chosen as reference, and analysed by EcoTILLING (Figure 3). Polymorphisms were observed in introns and exons, but only polymorphisms in exons were recorded. Six polymorphic sites were identified. Exons 1 and 5 contained 4 and 2 polymorphisms, respectively. No polymorphism was observed in exons 2, 3 and 4. Considering polymorphisms, we classified the accessions in six different haplotypes, named H.0 to H.5 (Table 1). Ninety seven accessions showed no polymorphisms in comparison to the reference, and were classified as haplotype H.0. In contrast, 23 accessions showed polymorphisms and were grouped in 5 different haplotypes, H1 to H.5 (Table 1). The most frequent haplotypes, apart from H.0, were H.1, observed for 7 accessions and corresponding to a polymorphism in exon 1, and H.3, observed for 5 accessions and corresponding to two polymorphisms in exon 1 (Table 1). H3 likely derives from H.2 as both haplotypes have in common one polymorphism (G186T) (Table 1). We demonstrated previously that *nsv *codes for an allele carrying a single nucleotide polymorphism (SNP) in exon 5 of *eIF4E *(position 683 from the start codon), and that this SNP is responsible for resistance to MNSV [18]. To estimate the frequency of the *nsv *allele, we analysed further by EcoTILLING the *Cucumis *spp. collection. Exon 5 was PCR amplified and heteroduplex DNAs were generated using accession PI 161375, homozygous for *nsv*, as reference. In this analysis, no cleaved product in exon 5 was observed from individuals of the H.4 haplotype and, thus, the *nsv *allele is represented by four accessions among the 120 tested.

**Table 1:** Classification of *Cucumis *spp. accessions according to their haplotype in EcoTILLING of *eIF4E*^a^

**Haplotype**	**No. of polymorphisms**	**Exon^**b**^/polymorphism^**c**^**	**Amino acid change**	**Accessions**^**d**^
H.0	-^e^	-	-	Védrantais and other 97 accessions
H.1	1	1/T81-A	Silent	C-087, C-163, C-182, C-707, C-840, C-841, C-842
H.2	1	1/G186-T	Silent	C-012, C-110, C-262, C-732
H.3	2	1/G186-T	Silent	C-035, C-204, C-205, C-492, WMR-29
		1/C243-T	Silent	
H.4	1	5/T683-A	Leu228-His	C-046, C-178, C-512, PI 161375
H.5	1	5/G690-A	Silent	C-105, C-117, C-759

^a ^Polymorphisms were observed in comparison with the cultivar Védrantais [18].

^b ^Exon harboring the SNPs/^c ^Position of the SNPs are given in reference to the translational start site.

^d ^Accessions are referred to by Estación Experimental "La Mayora" code's.

^e ^A dash indicates that no polymorphisms were identified.

### Variation of eIF4E versus virus susceptibility

The precise position and the nature of identified polymorphisms were determined by sequencing PCR products comprising exons 1 and 5 for all accessions from haplotypes H.1 to H.5 (except PI 161375). This also served to confirm that EcoTILLING was precise enough to localise polymorphisms in exons. Accessions of the same haplotype in EcoTILLING exhibited the same nucleotide change(s) (Table 1). Only nucleotide change T683-A of accessions of the H.4 haplotype was non-silent and corresponded to amino acid change Leu228-His. Therefore, a high degree of conservation of the eIF4E protein was observed. Significantly, all accessions of the H.4 haplotype were resistant to MNSV-Mα5, whereas all other accession grouped in haplotypes different to H.4 were susceptible to this virus (Table 2). Thus, a perfect correlation was found between amino acid change at position 228 of the eIF4E protein and resistance to MNSV-Mα5.

**Table 2:** *Cucumis *spp. accessions identified as potential sources of resistance^a ^and their eIF4E factors as characterised by EcoTILLING

			**Potential source of resistance to**^**b**^						
**Accession**	**Haplotype in EcoTILLING**	**Amino acid at position 228**	MNSV-Mα5	MNSV-264	CVYV	CMV	PRSV	WMV	ZYMV

C-019	H.0	His	S^c^	S	S	S	**R**	S	S
C-641	H.0	His	S	S	S	S	S	**R**	S
C-762	H.0	His	S	S	S	S	**R**	S	S
C-921	H.0	His	S	S	S	S	**R**	S	S
C-087	H.1	His	S	S	S	S	S	**R**	S
C-205	H.3	His	S	S	**R**	S	S	**R**	**R**
PI 161375	H.4	**Leu**	**R**	S	-	**R**	**R**	S	S
C-046	H.4	**Leu**	**R**	S	S	-	-	-	-
C-178	H.4	**Leu**	**R**	S	S	S	S	S	S
C-512	H.4	**Leu**	**R**	S	S	S	S	S	S
C-105	H.5	His	S	S	S	S	S	**R**	S
C-759	H.5	His	S	S	S	S	**R**	S	S

^a ^As identified by Díaz et al. [32] and in this work.

^b ^*Melon necrotic spot virus *(MNSV) strains Mα5 and 264, *Cucumber vein yellowing virus *(CVYV), *Cucumber mosaic virus *(CMV), *Papaya ringspot virus *strain W (PRSV-W), *Watermelon mosaic virus *(WMV) and *Zucchini yellow mosaic virus *(ZYMV).

^c ^S and **R **indicate susceptible and resistant accessions, respectively.

In addition to MNSV and CVYV, most of the accessions characterised here have been tested also for their susceptibility to CMV, *Papaya ringspot virus *strain W (PRSV-W), WMV and ZYMV [32]. Table 2 also includes accessions identified by Díaz et al. [32] as potential sources of resistance to these viruses. Except for the above mentioned case of MNSV, no correlation was found between variation of eIF4E and virus resistance (Table 2).

### Characterization of new *eIF4E *resistance alleles

**Figure 4:**
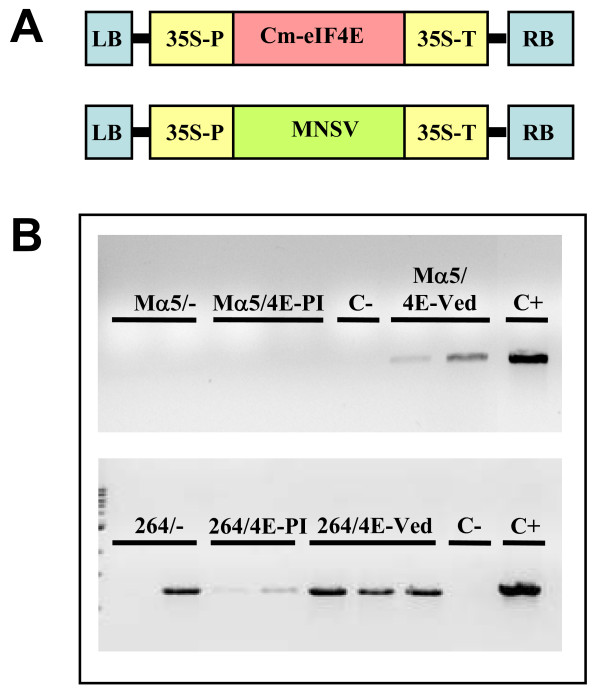
**Biolistic transient expression assay of *Cm-eIF4E-Ved *in *C. zeyheri***. (*A*) Schematic structure of MNSV and Cm-eIF4E constructs used in the transient expression assay. cDNAs were cloned into the binary vector pBIN61 between left (LB) and right (RB) borders of the *Agrobacterium *Ti plasmid. The 35S promoter and terminator are indicated as 35S-P and 35S-T, respectively. (*B*) RT-PCR detection of MNSV accumulation in bombarded leaves. pBMα5 (Mα5) and pB264 (264) constructs were bombarded separately and in combination with pB4E-PI (4E-PI) or pB4E-Ved (4E-Ved) into leaves of *C. zeyheri*. Two to three independent samples were included in the gel showed. Virus accumulation was assessed using RT-PCR two days post bombardment. C+ and C- indicate positive and negative controls of RT-PCR, respectively. C+ corresponds to leaves from susceptible melon bombarded with pBMα5 and pB264. In C-, RT-PCR was carried out with RNA from non-inoculated *C. zeyheri *leaves.

The only accession found to be resistant to both MNSV-Mα5 and MNSV-264 during the phenotypic screening was C-277 (*C. zeyheri*). *C. zeyheri eIF4E *(*Cz-eIF4E*) exons were PCR amplified and sequenced. Sequence comparisons showed that *Cz-eIF4E *exon 5 showed no variation with respect to *Cm-eIF4E-Ved*, the melon allele conferring susceptibility to MNSV [18]. Interestingly, exon 1 showed 5 polymorphisms able to give rise to 5 non-conservative amino acid changes. Given the implication of eIF4E of diverse species in virus susceptibility [6], we hypothesized that *Cz-eIF4E *could mediate *C. zeyheri *susceptibility to MNSV as *Cm-eIF4E *mediates melon susceptibility to this virus [18]. Our previous experience indicated that the co-expression of the melon susceptibility allele with the non-resistance breaking strain of MNSV in melon resistant plants indeed complements virus accumulation [18]. Therefore, we carried out a functional analysis based on the prediction that the co-expression of the susceptibility allele of *Cm-eIF4E *together with MNSV in *C. zeyheri *plants would complement virus accumulation. Appropriate DNA constructs (Figure 4A) [18] were bombarded into leaves of *C. zeyheri *plants and virus accumulation was assessed at 2 days post bombardment. In the MNSV-Mα5 case, we could not detect the accumulation of MNSV when it was bombarded alone or in combination with the melon resistance allele, but it was detected when it was bombarded together with the melon susceptibility allele (Figure 4B). In the MNSV-264 case, we detected the presence of the virus when it was bombarded alone, indicating that this strain can multiply, at least locally, in *C. zeyheri *tissues (Figure 4B). Notably, MNSV-264 accumulation seemed to be stimulated when it was co-bombarded with the melon susceptibility allele (Figure 4B).

## Discussion

### Use of EcoTILLING as a polymorphism discovery tool in melon

We have adapted and set up for the first time EcoTILLING in melon. This technology was initially used to characterise the variability of 5 genes within a collection of *Arabidopsis *ecotypes [30]. Then, it has been successfully used in analyses of the natural variability of wild populations of *Populus trichocarpa *[31], in the identification of allelic variation in resistance genes of barley [36] and it is being used for genotyping in other species [37]. Used in combination with sequencing, EcoTILLING is a very cost-effective technology: once polymorphisms are identified by EcoTILLING, individuals can be grouped according to haplotype and only interesting haplotypes, and/or representatives from each haplotype, can be sequenced; in addition, EcoTILLING allows the approximate location of the polymorphism within the locus studied and, therefore, restricts the necessity of sequencing the complete locus but only regions around the polymorphism. In our case, these reasons together with the low number of different haplotypes found have reduced in more than 90% the number of sequencing reactions potentially required to characterise the variability of *eIF4E *in our collection of melon accessions. Due to the limited number of accessions characterised in this work, pooling DNA from individual accessions [38] was not necessary. We expect that pooling would be feasible for *C. melo *accessions, but probably more difficult to apply when including wild melon relatives. In fact, one of the major problems that we have encountered is the difficulty in PCR amplifying eIF4E DNA from melon wild relatives, probably caused by misspriming. Once solved this problem, EcoTILLLING can be a potent tool for genetic analyses such as the study of heterozygosity in wild species, as it has been done for *Populus trichocarpa *[31].

### Variation in eIF4E *versus *virus susceptibility

Factor eIF4E is highly conserved in eukaryotes. The diversity found among factors from different organisms mainly resides at the amino-terminus of the protein, a region which may even have quite different lengths and which seems not to be directly involved in cap-binding [39-41]. In agreement with these data, we have found a very low diversity among *Cucumis *eIF4E. Taking into consideration results from the characterization of *Cz-eIF4E*, the amino-terminus of *Cucumis *eIF4E appears to be also the region where amino acid changes accumulate preferentially. However, our EcoTILLING results in *C. melo *uncover just one amino acid change, located at the very carboxy-terminus of the protein. Moreover, this change perfectly correlated with resistance to MNSV-Mα5, a result coincident with our previous observations [18]. The eIF4E carboxy-terminus, though outside of the cap-binding pocket, seems to have a critical role for functional regulation of cap binding through interactions with nucleotides downstream the cap [42]. MNSV RNA is uncapped, and our data indicate that a short non-coding region at the 3'-end of the viral RNA (virulence determinant) is critical for the outcome of the melon/MNSV interaction controlled by *nsv*, which encodes melon eIF4E [18]. A direct interaction between the virulence determinant and the eIF4E carboxy-terminus probably controls translation initiation of MNSV RNAs (Truniger, Nieto and Aranda, unpublished) and, thus, multiplication of the virus.

Interestingly, accessions used in this work have been previously tested for their susceptibility to CMV, PRSV-W, WMV and ZYMV, and potential sources for resistance to these viruses have been identified [32]. For example, the accession C-105 (TGR-1551) has been described as a potential source of resistance to WMV and to CMV and ZYMV aphid transmission [32,43] and the genetics of C-105 resistance to WMV has been characterised in detail [44]. However, our work has shown that all potential sources of resistance that have been analysed here, except those resistant to MNSV, have identical eIF4E proteins. It may be that the expression of eIF4E in resistant accessions is somehow altered through mutations in control regions of the gene, but this possibility seems to be unlikely given the critical role that this protein has in general translation initiation. Therefore, other factors, including translation initiation factors different than eIF4E, could control these resistances. The case of PI 161375 constitutes another interesting example. This accession exhibited resistance to MNSV, CMV and PRSV. It would be possible that the mutation Leu228-His in eIF4E controlling MNSV resistance also controls resistance to the other two viruses. However, this is unlikely, as accessions C-178 and C-512, with the same mutation, are fully susceptible to CMV and PRSV. Therefore, different or additional factors (i. e. molecular interactors and/or genetic loci) must be involved in the PI 161375 resistances to CMV and PRSV.

### New *eIF4E *alleles for MNSV resistance

Multiallelic, recessive resistance against plant viruses seems to be frequent (e.g. [22]), therefore we hypothesized that screenings to uncover the natural diversity of *eIF4E *might contribute to the discovery of new resistance alleles that can be incorporated into resistance breeding programs. However, in the case of MNSV resistance studied here, all *C. melo *accessions resistant to MNSV-Mα5 corresponded to a unique genetic type, and none of the *C. melo *accessions analysed here were resistant to MNSV-264. Nevertheless, we identified one melon wild relative accession (C-277), corresponding to *C. zeyheri*, that was resistant to both MNSV strains. Significantly, the analysis of the *Cz-eIF4E *sequence showed 5 polymorphisms in exon 1 that result into 5 non-conservative amino acid changes located at the amino-terminus of the protein; none of these changes had a correspondence with the SNP responsible for the change of MNSV susceptibility in melon [18]. Therefore, we hypothesized that *Cz-eIF4E *could be a new allele for resistance to MNSV. The complementation experiments described in this paper allow speculation in this regard. In *nsv *resistant melons, co-bombardment of MNSV-Mα5 together with the melon susceptibility allele results in virus multiplication [18]. Similarly, here we observed that when *C. zeyheri *plants are co-bombarded with MNSV-Mα5 and the melon susceptibility allele, virus multiplication could be detected, whereas co-bombardment with the melon resistance allele does not result in virus multiplication. Assuming that *Cz-eIF4E *has an expression pattern equivalent to that of *Cm-eIF4E*, these results strongly suggest that *Cz-eIF4E *is unable to contribute to MNSV-Mα5 multiplication and, therefore, Cz-eIF4E may constitute the factor controlling resistance to MNSV-Mα5 in *C. zeyheri *plants. The situation seems to be different for MNSV-264. On the one hand, there is an apparent contradiction between the results of the bombardment experiments and the phenotypic screening: bombardment of *C. zeyheri *plants with MNSV-264 showed that this viral strain can accumulate in inoculated leaves of *C. zeyheri *plants, while results of the phenotypic screenings indicated that this accession is resistant to MNSV-264. This discrepancy may be due to differences in the inoculation and detection methods used in both assays or, alternatively, MNSV-264 movement might be restricted to the initial infection foci in *C. zeyheri *plants. On the other hand, bombardment experiments have suggested that the presence of the *C. melo *eIF4E susceptibility allele stimulates the MNSV-264 multiplication in *C. zeyheri *tissues. To be fully understood, results concerning MNSV-264 bombardments on *C. zehyeri *tissues require additional experiments.

## Conclusion

The low variability found for melon eIF4E, together with data on the importance of eIF4E as a virus susceptibility factor [6], recommend approaching the generation of new eIF4E alleles through mutagenesis. High throughput identification of melon *eIF4E *mutants should be feasible, and TILLING could be an appropriate technology for this purpose. Our data has also pointed to the importance of considering additional candidate genes as susceptibility factors: resistance of *Cucumis *spp. accessions to different viruses seemed not to rely uniquely on eIF4E. Thus, identification of new susceptibility factors in model species, together with phenotypic screenings of the natural species diversity, are activities of the outmost importance to identify new sources of virus resistance.

## Methods

### Plant and virus materials

*Cucumis *accessions were obtained from the germplasm collection maintained at Estación Experimental "La Mayora"- CSIC (Málaga, Spain) and included 135 *C. melo *land races and traditional cultivars as well as 12 accessions of wild relatives (1 accession of *C. myriocarpus*, 1 of *C. metuliferus*, 2 of *C. africanus*, 1 of *C. zeyheri*, 1 of *C. dipsaceus*, 1 of *C. prophetarum*, 1 of *C. meeusei*, 1 of *C. anguria*, 1 of *C. ficifolius *and 2 of *Cucumis *spp.). Among the *C. melo *accessions there were two controls for which virus susceptibility has already been tested: cv. Rochet, which is susceptible to MNSV and CVYV, and cv. Planters Jumbo, resistant to all MNSV isolates tested except to MNSV-264 [26]. Accession numbers and geographical origins of accessions are listed in Additional file 1.

The viral isolates used in this study were MNSV-Mα5 [33], MNSV-264 [26] and CVYV-AlLM [34].

### Inoculation and evaluation procedures

Plants of each accession were inoculated mechanically by rubbing carborundum-dusted cotyledons with extracts of infected plant material. Infectious extracts were prepared from susceptible *C. melo *cv. Rochet plants inoculated 15 days earlier, by grinding 0.1 g of young symptomatic tissue in 2 ml of 30 mM Na_2_HPO_4_, 0.2% (wt/vol) Na-diethyldithiocarbamate, in the CVYV case, and 10 mM K_2_HPO_4_-KH_2_PO_4 _(pH 7), in the MNSV case. Plants were inoculated at the fully expanded cotyledons growth stage. For CVYV, plants were inoculated a second time five days after the first inoculation. Presence or absence of virus symptoms was recorded for each test plant at 7, 15 and 25 days after inoculation. Then, in two symptomatic plants per accession and in all asymptomatic plants or with no clear symptoms, presence of CVYV or MNSV was analysed by molecular hybridisation in tissue prints of cross sections of petioles from young leaves [45] using probes decribed in [33,34]. Ten plants per accession and virus combination were normally used for inoculations. Only those accessions in which the 10 plants tested negative were considered resistant. Accessions that rated as resistant were tested at least twice for confirmation. Plants were maintained after inoculations in an insect-proof glasshouse at aproximately 25°C day, 18°C night, 45–85% relative humidity and 16 h day lenght, with light supplementation when needed.

### DNA extractions and screening for polymorphisms

Genomic DNA of accessions used in EcoTILLING was prepared from young leaves of plants grown in a growth chamber at 25°C day, 19°C night, 50% relative humidity and 16-h day length. Four discs of 1 cm diameter obtained from 4 individual plants were used per accession. DNA was extracted using the DNeasy 96 Plant DNA Purification Kit (Qiagen, Hilden, Germany) according to the manufacturer's protocol. Polymerase Chain reaction (PCR) and EcoTILLING were performed as described by [30] with minimal modifications, using 96 well plates. PCR was carried out in a final volume of 25 μL, using 5–10 ng/μL of template DNA and three primer pairs: to amplify exon 1, 5'-GAGGGCGGTGCCATTCTTCTTCGG-3' (Full-cDNA5'-F) and 5'-TCCCTAAATCGAACCAAGAAACGCC-3' (Intron1-R); to amplify exons 2 to 5, 5'-TGCTTGGCTGTTAATTTATCTCTGC-3' (Intron1-F) and 5'-GTCAAGTACAGAACAAGAATCTGAG-3' (Full-cDNA3'-R); and to amplify exon 5, 5'-TACATGCGGCTGTATAAATTTCAGC-3' (Intron4-F) and Full-cDNA3'-R (Figure 2). Exon 5 was specifically amplified pursuing maximum accuracy, as it is here where a polymorphism controlling melon susceptibility to MNSV has been identified [18]. Primers were designed based on the sequences of *Cm-eIF4E *genomic DNAs determined for melon cv. Védrantais (susceptible to MNSV-Mα5) and accession PI 161375 (resistant to MNSV-Mα5) [18]. All forward primers were 5'-end IRDye 700 labelled (red) and reverse primers 5'-end IRDye 800 labelled (green) (MWG-Biotech, Ebersberg, Germany). PCR products were checked by agarose gel electrophoresis and then, 3 μL (approximately 20 ng) of each PCR product to be tested were mixed with the same amount of reference DNA, which was in all cases the equivalent fragment amplified from the melon cv. Védrantais. Additionally, for amplification products corresponding to exon 5, the fragment amplified from the melon accession PI 161373 was also used as reference. The mixture was denatured at 94°C for 3 min and reannealed using a temperature gradient of 0.1°C/s up to 8°C to allow formation of heteroduplexes. PCR products were digested with a mismatch specific endonuclease, *ENDO-1*, in a final volume of 30 μL which contained 6 μL of the mixed DNAs, 3 μL the 10× *ENDO-1 *buffer (1M HEPES, 1M MgSO4, 10% Triton X-100 and 2 M KCl) and 0.03 μL of pure *ENDO-1 *(Bendahmane, unpublished results). Digestion was incubated at 42°C for 20 min and stopped by adding 5 μL of EDTA 0.15 M. The DNA was purified by passage through G50 Sephadex (S-G50; GE Healthcare Life Sciences, Little Chalfont, UK). Five μL of Formamide Loading Dye (GE Healthcare Life Sciences) were added to each DNA sample and the loading mixture was concentrated for 50 min at 65°C up to a volume of approximately 5 μL. Samples (0.6 μL) were run on a LI-COR sequencing gel (DNA LI-COR 4300; LI-COR Biosciences, Lincoln, Nebraska, USA) with a 0.4 mm, 96-well comb. Gels were run at 1500 V/40 W/45°C for 2–4 h. Analyses of the gel images were carried out manually using Adobe Photoshop. When a putative polymorphism was found by EcoTILLING, the corresponding DNA fragment was sequenced for verification.

### Characterization of *Cz-eIF4E*

Exons 1 and 5 of *Cz-eIF4E *were amplified using 5–10 ng/μL of gDNA and the same primer combinations as described above. Annealing temperature was decreased to 50°C. PCR products were sequenced and a new primer pair [5'-CAGGCCACCTGGGGTGCGTCTATTCGACCG-3' (277-F); 5'-AGTATCCTCCTCCCACGCCACTAGAAACCG-3' (277-R)] was designed in the non-coding regions upstream exon 2 and downstream exon 5 of *Cz-eIF4E *specific sequence. A nested PCR was carried out using the primer combinations Full-cDNA5'-F/Full-cDNA3'-R and 277-F/277-R. Exons 2, 3 and 4 were sequenced from the product of the nested-PCR.

For complementation assays, constructs expressing *Cm-eIF4E-Ved*, *Cm-eIF4E-PI*, MNSV-Mα5 and MNSV-264 were used [18]. The constructs derived from MNSV-Mα5 and MNSV-264 were referred to as pBMα5 and pB264, respectively. The *Cm-eIF4E *constructs derived from resistant (PI 161375) and susceptible (Védrantais) genotypes were referred to as pB4E-PI and pB4E-Ved, respectively (Figure 4A). Twenty μg of plasmid DNA from viral and *Cm-eIF4E *expression vectors were mixed in a ratio of 1/3 before being coated to 1.0 Micron Gold particles (BioRAD, Hercules, CA, USA) as described previously [18]. Detached leaves from 6-week-old plants were bombarded with the gold particles coated with plasmid DNAs, using the Biolistic PDS-1000/He System (BioRAD, Hercules, CA, USA). The leaves were incubated in moistened Petri dishes at 25°C for 48 hours. RNA extraction (TRIzol Reagent, Invitrogen, Carlsbad, CA, USA) was performed and then analysed for virus accumulation using RT-PCR. The primer Seq3'α5-R (5'-GGAACAAACTTGGAGAGTATACAAAGAG-3') was used to synthesize the first cDNA strand and Seq1-F (5'-CCCATCAAAACACGCAAACTGTATTGTC-3') and Seq1-R (5'-ACACTGAAACCCGAATTGTCTCCAGTG-3') primers were used for PCRs.

## Authors' contributions

Cristina Nieto performed most of the analyses and participated in the design of the study. Florence Piron and Marion Dalmais contributed to the implementation of the EcoTILLING technique and processed the samples. Cristina F. Marco, Ma Luisa Gómez-Guillamón, Verónica Truniger, Enrique Moriones and Pedro Gómez are responsible for providing and maintaining the germplasm and for phenotypic screenings. Jordi Garcia-Mas contributed to project conception and manuscript drafting. Miguel A. Aranda contributed to project conception, co-supervised the study and wrote the manuscript. Abdelhafid Bendahmane is the principal investigator, conceived the study and participated in its design and coordination. All authors read and approved the final manuscript.

## Supplementary Material

Additional file 1 ***Cucumis *spp. accessions analyzed by EcoTILING**. Accession number, taxonomic denomination, geographical origin and results of CVYV and MNSV susceptibility analyses are given for all accessions characterized in this work. Click here for file
