# Supplementary material for: EcoTILLING for the identification of allelic variants of melon eIF4E, a factor that controls virus susceptibility
Source: BMC Plant Biol. 2007 Jun 21;7:34. doi: 10.1186/1471-2229-7-34 (PMC1914064; doi:10.1186/1471-2229-7-34)
Supplement: Additional file 1 — Cucumis spp. accessions analyzed by EcoTILING. Accession number, taxonomic denomination, geographical origin and results of CVYV and MNSV susceptibility analyses are given for all accessions characterized in this work. [file 1471-2229-7-34-S1.pdf]

**Additional file 1: *Cucumis* ssp. accessions analyzed by EcoTILLING.** Accession number, taxonomic denomination, geographical origin and results of CVYV and MNSV susceptibility analyses are given for all accessions characterized in this work.

| Accession number[a] | Species | Origin | EcoTILLING haplotype | CVYV[b] | MNSV-Mα5 | | MNSV-264 | |
|---|---|---|---|---|---|---|---|---|
| | | | | | Les/cot [c] | Sys. Symp.[d] | Les/cot [e] | Sys. Symp.[f] |
| **C-001** | *Cucumis melo* | Spain | H.0 | 10/10 | 5 | 30% | 18 | 15% |
| **C-002** | *Cucumis melo* | Spain | H.0 | 6/10 | 8 | 100% | >50 | 0% |
| **C-008** | *Cucumis melo* | Spain | H.0 | 10/10 | 2 | 0% | 2 | 0% |
| **C-010** | *Cucumis melo* | Spain | H.0 | -[g] | 8 | 15% | >50 | 0% |
| **C-012** | *Cucumis melo* | Mexico | H.2 | 6/10 | 1 | 0% | 1 | 0% |
| **C-019** | *Cucumis melo* | Spain | H.0 | 9/10 | 8 | 0% | >50 | 0% |
| **C-025** | *Cucumis melo* | Portugal | H.0 | 10/10 | 21 | 0% | 9 | 0% |
| **C-029** | *Cucumis melo* | USA | H.0 | 11/19 | 3 | 0% | 5 | 0% |
| **C-035** | *Cucumis melo* | Spain | H.3 | 7/10 | 10 | 100% | 10 | 100% |
| **C-040** | *Cucumis melo* | Japan | H.0 | 10/10 | 20 | 0% | 18 | 0% |
| **C-046** | *Cucumis melo* | USA | H.4 | 9/10 | 0 | 0 | 7 | 30% |
| **C-048** | *Cucumis melo* | Israel | H.0 | 10/10 | 7 | 0% | 3 | 0% |
| **C-053** | *Cucumis melo* | Spain | H.0 | 9/10 | 6 | 100% | 15 | 100% |
| **C-055** | *Cucumis melo* | Spain | H.0 | 10/10 | 7 | 50% | 5 | 0% |
| **C-058** | *Cucumis melo* | Spain | H.0 | 10/10 | 4 | 0% | 3 | 0% |
| **C-062** | *Cucumis melo* | Spain | H.0 | 10/10 | 7 | 25% | 2 | 0% |
| **C-065** | *Cucumis melo* | Spain | H.0 | 10/10 | 4 | 13% | 3 | 0% |
| **C-066** | *Cucumis melo* | Spain | H.0 | 10/10 | 12 | 0% | 10 | 0% |
| **C-069** | *Cucumis melo* | Spain | H.0 | - | 6 | 0% | >50 | 0% |
| **C-070** | *Cucumis melo* | Spain | H.0 | 10/10 | 2 | 0% | 3 | 0% |
| **C-071** | *Cucumis melo* | Spain | H.0 | 10/10 | 3 | 16% | 3 | 0% |
| **C-072** | *Cucumis myriocarpus* | Unknown | No amplification | 8/10 | 3 | 0% | 2 | 0% |
| **C-075** | *Cucumis melo* | Spain | H.0 | 10/10 | 3 | 28% | 5 | 14% |
| **C-076** | *Cucumis metuliferus* | Unknown | No amplification | 5/5 | 2 | 0% | 17 | 0% |
| **C-078** | *Cucumis melo* | Spain | H.0 | 8/10 | 7 | 0% | 8 | 0% |
| **C-087** | *Cucumis melo* | Unknown | H.1 | 7/10 | 3 | 0% | 8 | 0% |
| **C-098** | *Cucumis melo* | Spain | H.0 | - | 6 | 71% | >30 | 0% |
| **C-105** | *Cucumis melo* | Zimbabwe | H.5 | 8/10 | >30 | 85% | 25 | 60% |
| **C-110** | *Cucumis melo* | Spain | H.2 | 12/20 | 6 | 43% | 15 | 43% |
| **C-117** | *Cucumis melo* | Zimbabwe | H.5 | 9/10 | 6 | 25% | 12 | 0% |

| Accession number[a] | Species | Origin | EcoTILLING haplotype | CVYV[b] | MNSV-Mα5 | | MNSV-264 | |
|---|---|---|---|---|---|---|---|---|
| | | | | | Les/cot [c] | Sys. Symp.[d] | Les/cot [e] | Sys. Symp.[f] |
| **C-125** | *Cucumis melo* | Zimbabwe | H.0 | 9/10 | 4 | 67% | 6 | 0% |
| **C-160** | *Cucumis melo* | Ex URSS | H.0 | 10/10 | 5 | 71% | >50 | 0% |
| **C-163** | *Cucumis melo* | Ex URSS | H.1 | 10/10 | 20 | 0% | 9 | 0% |
| **C-169** | *Cucumis melo* | Greece | H.0 | 10/10 | 9 | 0% | 4 | 0% |
| **C-172** | *Cucumis melo* | Italy | H.0 | 8/8 | <1 | 0% | 5 | 0% |
| **C-178** | *Cucumis melo* | USA | H.4 | 9/10 | 0 | 0% | 3 | 0% |
| **C-182** | *Cucumis melo* | China | H.1 | 10/10 | 5 | 0% | 6 | 0% |
| **C-187** | *Cucumis melo* | Germany | H.0 | 10/10 | 10 | 14% | 6 | 0% |
| **c-192** | *Cucumis melo* | India | H.0 | 9/10 | 6 | 14% | 5 | 0% |
| **C-194** | *Cucumis melo* | Ex URSS | H.0 | 9/10 | 6 | 0% | 5 | 0% |
| **C-200** | *Cucumis melo* | Mexico | H.0 | 10/10 | 10 | 0% | 10 | 0% |
| **C-204** | *Cucumis melo* | Libya | H.3 | 10/10 | 3 | 0% | 3 | 11% |
| **C-205** | *Cucumis africanus* | Unknown | H.3 | 0/10 | <1 | 0% | <1 | 0% |
| **C-219** | *Cucumis melo* | Spain | Not tested | 10/10 | 10 | 50% | 18 | 0% |
| **C-225** | *Cucumis melo* | Spain | H.0 | 10/10 | 1 | 15% | 3 | 0% |
| **C-229** | *Cucumis melo* | Spain | H.0 | 9/10 | 5 | 0% | 5 | 0% |
| **C-233** | *Cucumis melo* | Spain | H.0 | 10/10 | 3 | 15% | 2 | 0% |
| **C-235** | *Cucumis melo* | Spain | H.0 | 10/10 | 5 | 17% | 3 | 0% |
| **C-241** | *Cucumis melo* | Spain | H.0 | 10/10 | 5 | 57% | 5 | 20% |
| **C-243** | *Cucumis melo* | Spain | H.0 | 10/10 | 9 | 16% | 8 | 16% |
| **C-245** | *Cucumis melo* | Spain | H.0 | 10/10 | 7 | 15% | 6 | 0% |
| **C-247** | *Cucumis melo* | Libya | H.0 | 10/10 | 3 | 0% | 2 | 0% |
| **C-250** | *Cucumis melo* | Spain | H.0 | 10/10 | 15 | 43% | 8 | 0% |
| **C-262** | *Cucumis melo* | Libya | H.2 | 10/10 | 6 | 57% | 5 | 29% |
| **C-269** | *Cucumis melo* | China | H.0 | 10/10 | 6 | 0% | 6 | 0% |
| **C-271** | *Cucumis melo* | Spain | H.0 | - | 9 | 28% | >50 | 50% |
| **C-273** | *Cucumis melo* | USA | Not tested | 10/10 | 8 | 0% | >50 | 0% |
| **C-277** | *Cucumis zeyheri* | Unknown | No amplification | 0/10 | 0 | 0% | 0 | 0% |
| **C-278** | *Cucumis melo* | Spain | H.0 | - | 6 | 83% | 8 | 43% |
| **C-300** | *Cucumis melo* | Spain | H.0 | 7/10 | 2 | 15% | 3 | 0% |
| **C-305** | *Cucumis melo* | Spain | H.0 | - | 5 | 43% | >50 | 0% |
| **C-308** | *Cucumis melo* | Spain | H.0 | - | 8 | 40% | 8 | 0% |
| **C-311** | *Cucumis melo* | Beluchistan | H.0 | 10/10 | >50 | 67% | 6 | 0% |

| Accession number[a] | Species | Origin | EcoTILLING haplotype | CVYV[b] | MNSV-Mα5 | | MNSV-264 | |
|---|---|---|---|---|---|---|---|---|
| | | | | | Les/cot [c] | Sys. Symp.[d] | Les/cot [e] | Sys. Symp.[f] |
| **C-317** | *Cucumis melo* | Spain | H.0 | - | 5 | 0% | 50 | 0% |
| **C-319** | *Cucumis melo* | Spain | H.0 | - | 15 | 100% | 50 | 100% |
| **C-323** | *Cucumis melo* | Spain | H.0 | - | 7 | 15% | 50 | 0% |
| **C-326** | *Cucumis melo* | Spain | H.0 | - | 4 | 43% | >30 | 0% |
| **C-330** | *Cucumis melo* | Spain | H.0 | - | 14 | 100% | >50 | 86% |
| **C-333** | *Cucumis melo* | Spain | H.0 | - | 4 | 100% | 8 | 28% |
| **C-340** | *Cucumis melo* | Beluchistan | H.0 | - | >50 | 33% | >50 | 10% |
| **C-343** | *Cucumis melo* | Spain | H.0 | - | 2 | 33% | 4 | 43% |
| **C-344** | *Cucumis melo* | Spain | H.0 | - | 10 | 72% | 25 | 0% |
| **C-352** | *Cucumis melo* | Spain | H.0 | - | 5 | 14% | >50 | 0% |
| **C-406** | *Cucumis melo* | Spain | H.0 | 10/10 | 8 | 29% | 6 | 0% |
| **C-409** | *Cucumis melo* | Spain | H.0 | 10/10 | 7 | 0% | 5 | 0% |
| **C-411** | *Cucumis melo* | Spain | H.0 | 10/10 | 8 | 15% | 10 | 15% |
| **C-412** | *Cucumis melo* | Spain | H.0 | 10/10 | 5 | 28% | 2 | 0% |
| **C-426** | *Cucumis melo* | Spain | H.0 | - | 12 | 15% | 7 | 0% |
| **C-447** | *Cucumis melo* | USA | H.0 | 10/10 | 15 | 43% | 12 | 15% |
| **C-451** | *Cucumis melo* | Ex Yugoslavia | H.0 | 9/10 | >50 | 17% | 23 | 0% |
| **C-457** | *Cucumis melo* | Ex Yugoslavia | H.0 | - | 19 | 57% | >50 | 14% |
| **C-488** | *Cucumis melo* | Spain | H.0 | 9/10 | >50 | 15% | 3 | 13% |
| **C-489** | *Cucumis melo* | Spain | H.0 | 10/10 | 2 | 15% | 4 | 0% |
| **C-492** | *Cucumis melo* | Spain | H.3 | 10/10 | 7 | 20% | >50 | 20% |
| **C-498** | *Cucumis melo* | Spain | H.0 | 10/10 | 5 | 50% | 6 | 0% |
| **C-512** | *Cucumis melo* | Spain | H.4 | 10/10 | 0 | 0% | 3 | 15% |
| **C-523** | *Cucumis melo* | Spain | H.0 | 10/10 | 8 | 0% | 7 | 0% |
| **C-538** | *Cucumis melo* | Spain | H.0 | 8/10 | 9 | 0% | 10 | 0% |
| **C-590** | *Cucumis dipsaceus* | Unknown | No amplification | 0/10 | 0 | 0% | <1 | 0% |
| **C-629** | *Cucumis melo* | Italy | H.0 | 8/10 | >50 | 20% | 10 | 20% |
| **C-630** | *Cucumis africanus* | Unknown | No amplification | 0/9 | <1 | 0% | <1 | 0% |
| **C-633** | *Cucumis prophetarum* | Unknown | Not tested | 0/10 | <1 | 15% | <1 | 0% |
| **C-635** | *Cucumis meeusii* | Unknown | No amplification | 10/10 | 0 | 0% | <1 | 0% |
| **C-636** | *Cucumis anguria* | Unknown | No amplification | 10/10 | 0 | 0% | <1 | 0% |
| **C-637** | *Cucumis ficifolius* | Unknown | No amplification | 7/10 | <1 | 15% | <1 | 0% |
| **C-641** | *Cucumis melo* | Zambia | H.0 | 8/10 | 3 | 0% | 2 | 0% |

| **Accession number[a]** | **Species** | **Origin** | **EcoTILLING haplotype** | **CVYV[b]** | **MNSV-Mα5** | | **MNSV-264** | |
|---|---|---|---|---|---|---|---|---|
| | | | | | **Les/cot [c]** | **Sys. Symp.[d]** | **Les/cot [e]** | **Sys. Symp.[f]** |
| **C-646** | *Cucumis melo* | Spain | H.0 | 10/10 | 9 | 44% | 4 | 0% |
| **C-701** | *Cucumis melo* | Pakistan | H.0 | 10/10 | 2 | 15% | 4 | 0% |
| **C-707** | *Cucumis melo* | Pakistan | H.1 | 10/10 | 7 | 33% | 6 | 15% |
| **C-719** | *Cucumis melo* | Ex Yugoslavia | H.0 | 10/10 | 4 | 17% | 2 | 0% |
| **C-726** | *Cucumis melo* | Spain | H.0 | 10/10 | >50 | 0% | 3 | 0% |
| **C-732** | *Cucumis melo* | Unknown | H.2 | 9/10 | 2 | 0% | 2 | 0% |
| **C-747** | *Cucumis melo* | Ex Yugoslavia | H.0 | 9/10 | <1 | 0% | <1 | 0% |
| **C-753** | *Cucumis sp.* | Unknown | H.0 | 9/10 | <1 | 15% | <1 | 15% |
| **C-755** | *Cucumis sp.* | Unknown | H.0 | 7/10 | <1 | 0% | <1 | 0% |
| **C-756** | *Cucumis melo* | Spain | H.0 | - | 11 | 71% | >50 | 0% |
| **C-757** | *Cucumis melo* | Spain | H.0 | - | >50 | 100% | >50 | 100% |
| **C-758** | *Cucumis melo* | Spain | H.0 | 9/10 | >50 | 33% | <1 | 0% |
| **C-759** | *Cucumis melo* | Spain | H.5 | 9/10 | 6 | 0% | 3 | 0% |
| **C-761** | *Cucumis melo* | Spain | H.0 | 10/10 | 3 | 33% | 10 | 0% |
| **C-762** | *Cucumis melo* | Spain | Not tested | 10/10 | 10 | 28% | 6 | 0% |
| **C-763** | *Cucumis melo* | Spain | H.0 | 10/10 | 6 | 15% | 1 | 0% |
| **C-765** | *Cucumis melo* | Spain | H.0 | 10/10 | 8 | 15% | 2 | 0% |
| **C-766** | *Cucumis melo* | Spain | H.0 | 10/10 | 5 | 0% | 3 | 0% |
| **C-767** | *Cucumis melo* | Spain | H.0 | 9/10 | 5 | 28% | 5 | 0% |
| **C-826** | *Cucumis melo* | Spain | H.0 | 9/10 | 6 | 12% | 3 | 0% |
| **C-827** | *Cucumis melo* | Spain | H.0 | - | 7 | 15% | 10 | 0% |
| **C-828** | *Cucumis melo* | Spain | H.0 | - | 3 | 0% | 5 | 0% |
| **C-829** | *Cucumis melo* | Spain | H.0 | - | 12 | 100% | >50 | 0% |
| **C-830** | *Cucumis melo* | Spain | H.0 | - | 9 | 100% | >50 | 0% |
| **C-831** | *Cucumis melo* | Spain | H.0 | - | 15 | 43% | 5 | 0% |
| **C-832** | *Cucumis melo* | Spain | H.0 | - | 15 | 13% | >50 | 0% |
| **C-834** | *Cucumis melo* | Spain | H.0 | 9/10 | 10 | 0% | 8 | 0% |
| **C-837** | *Cucumis melo* | Israel | H.0 | 8/10 | 5 | 0% | 7 | 0% |
| **C-839** | *Cucumis melo* | Israel | H.0 | 9/10 | 6 | 25% | 4 | 0% |
| **C-840** | *Cucumis melo* | Rusia | H.1 | 9/10 | 7 | 28% | 5 | 0% |
| **C-841** | *Cucumis melo* | Israel | H.1 | 10/10 | 5 | 57% | 3 | 0% |
| **C-842** | *Cucumis melo* | Israel | H.1 | 9/10 | 7 | 71% | 5 | 15% |
| **C-843** | *Cucumis melo* | Ukraine | Not tested | 10/10 | 4 | 25% | 3 | 13% |

| Accession number[a] | Species | Origin | EcoTILLING haplotype | CVYV[b] | MNSV-Mα5 | | MNSV-264 | |
|---|---|---|---|---|---|---|---|---|
| | | | | | Les/cot [c] | Sys. Symp.[d] | Les/cot [e] | Sys. Symp.[f] |
| **C-844** | *Cucumis melo* | Turkey | H.0 | 10/10 | 3 | 28% | 4 | 15% |
| **C-861** | *Cucumis melo* | Germany | Not tested | 9/10 | 1 | 0% | 3 | 0% |
| **C-862** | *Cucumis melo* | Greece | Not tested | 10/10 | 4 | 33% | 2 | 33% |
| **C-863** | *Cucumis melo* | Germany | Not tested | 10/10 | 4 | 25% | 4 | 0% |
| **C-864** | *Cucumis melo* | Russia | Not tested | 10/10 | <1 | 20% | <1 | 0% |
| **C-865** | *Cucumis melo* | Georgia | Not tested | 10/10 | <1 | 17% | <1 | 0% |
| **C-866** | *Cucumis melo* | Germany | Not tested | 10/10 | <1 | 17% | 6 | 0% |
| **C-868** | *Cucumis melo* | Ex URSS | Not tested | 10/10 | 4 | 0% | <1 | 0% |
| **C-869** | *Cucumis melo* | Mongolia | Not tested | 10/10 | 2 | 21% | 9 | 0% |
| **C-870** | *Cucumis melo* | Italy | Not tested | 10/10 | 12 | 28% | 7 | 0% |
| **C-872** | *Cucumis melo* | Tadshikistan | Not tested | 10/10 | <1 | 75% | 3 | 0% |
| **C-874** | *Cucumis melo* | Mongolia | Not tested | 8/10 | 8 | 25% | 2 | 0% |
| **C-875** | *Cucumis melo* | Irak | Not tested | 10/10 | 90 | 57% | >50 | 57% |
| **C-878** | *Cucumis melo* | Italy | Not tested | 5/10 | 2 | 100% | 2 | 12% |
| **C-880** | *Cucumis melo* | Georgia | Not tested | 10/10 | 5 | 0% | 3 | 0% |
| **C-881** | *Cucumis melo* | Georgia | Not tested | 10/10 | 15 | 15% | >50 | 28% |
| **C-883** | *Cucumis melo* | Canadá | Not tested | 10/10 | 5 | 0% | 10 | 8% |
| **C-887** | *Cucumis melo* | Albania | Not tested | 9/10 | 6 | 28% | 20 | 40% |
| **C-889** | *Cucumis melo* | Italy | Not tested | 10/10 | >50 | 57% | >50 | 15% |
| **C-921** | *Cucumis melo* | Spain | H.0 | 10/10 | 8 | 16% | 2 | 0% |
| **WMR-29** | *Cucumis melo* | - | H.3 | 10/10 | - | - | - | - |

[a]Accession numbers of germplasm collection of Estación Experimental "La Mayora"- CSIC (Málaga, Spain).
[b]Number of infected plants/ number of inoculated plants with CVYV at 15 dpi.
[c]Average number of local lesions / cotyledon after MNSV-Mα5 inoculation.
[d]Percentage of plants showing systemic symptoms with MNSV-Mα5 at 5 dpi.
[e]Average number of local lesions / cotyledon after MNSV-264 inoculation.
[f]Percentage of plants showing systemic symptoms with MNSV-264 at 5 dpi.
[g]A dash (-) indicates that no data is available for this particular accession or observation.
